# Current Status and Barriers of Ophthalmic Examination Services in Companion Animal Clinics in South Korea

**DOI:** 10.1155/vmi/9925114

**Published:** 2025-12-08

**Authors:** Jae-Won Kim, Holden Yoon Seung Kim, Kyeongmin Kim

**Affiliations:** ^1^ Corporate Research Institute, Laboratory for Sustainable Development, Inc., Seoul, 07331, Republic of Korea; ^2^ Planning Team, Laboratory for Sustainable Development, Inc., Seoul, 07331, Republic of Korea; ^3^ Department of Social Welfare, Graduate School, Seoul National University, Seoul, 08826, Republic of Korea, snu.ac.kr

**Keywords:** eye examination, fundus examination, South Korea, veterinary clinic, veterinary medicine, veterinary ophthalmology

## Abstract

**Objective:**

To investigate the current status of ophthalmic examinations, referral practices to specialized veterinary eye hospitals, and the associated challenges faced by veterinarians in South Korea.

**Procedures:**

A cross‐sectional online survey was conducted in South Korea from April 23 to May 6, 2024. A total of 114 veterinarians participated in the study.

**Results:**

Over 80% of respondents (*n* = 101) reported providing general ocular examinations, including vision testing and anterior segment examination. However, only about one‐third (*n* = 35) performed fundus examinations. The main barriers to performing fundus exams were the high cost of equipment (70.3%), lack of diagnostic confidence (48.3%), and perceived low clinical need (28.1%).

**Conclusion:**

While general ophthalmic services are widely available, the provision of fundic examinations remains limited. Addressing this gap may require increasing awareness of affordable diagnostic tools, enhancing veterinarians’ diagnostic confidence through education and training, and emphasizing the clinical importance of fundic examinations.

## 1. Introduction

In South Korea, pet ownership has increased significantly, rising from 17.2% in 2010 to 26.9% in 2019 [1]. Reflecting this growth, the domestic pet‐related market has expanded rapidly. According to projections, it will reach 21 trillion KRW(approximately $15.2 billion) by 2032 [2, 3]. This growing market has led to heightened expectations for high‐quality veterinary services and raised discussions about the need for more specialized care [4].

In South Korea, veterinarians often pursue advanced postgraduate training (such as master’s or doctoral degrees) for specialized practice. However, the country currently lacks a formal board certification system system under national law. Although veterinary specialty societies offer their own “injeungjeonmunui” certifications, these do not confer legal specialist status because they are not officially recognized by the government [5].

Despite the advanced training and the existence of these private certifications, the growing need for specialized veterinary services is poorly documented. There is a general lack of accessible data on how these services are actually provided in clinical practice, which has severely limited research in this area. As a result, fully understanding the landscape of specialized veterinary care remains a challenge, and empirical studies are scarce.

To address this gap in empirical data, this study collected survey data from veterinary clinics and hospitals across South Korea. In this study, we particularly focused on ophthalmic services, as veterinary ophthalmology represents a specialty field with increasing clinical relevance and significance. It is supported by relatively well‐established certification systems both domestically and internationally, reflecting growing need and clinical importance [6]. Ultimately, this study aims to inform the development of more accessible and specialized veterinary care in South Korea by analyzing the current provision of ophthalmic examinations, referral patterns to animal eye hospitals, and barriers faced by general practitioners in providing these services.

## 2. Materials and Methods

### 2.1. Ethical Statements

All survey participants provided written consent remotely prior to participation. The only personal information collected was mobile phone numbers, which were used solely for sending participation incentives. As the survey was initially conducted for market research purposes, a retrospective review by the Institutional Review Board (IRB) was performed. The study was granted an exemption by the Public Institutional Bioethics Committee under the Ministry of Health and Welfare in South Korea (IRB No. P01‐202406‐01‐012).

### 2.2. Study Population

The target population consisted of veterinarians practicing in South Korea.

### 2.3. Sample Selection

Respondents were recruited via personalized messages through social media platforms, including Naver TalkTalk , KakaoTalk, Instagram, and Naver Blogs. Using Naver Map, approximately 1000 veterinary hospitals and clinics were identified and contacted through direct messages or inquiry. About 10% responded and completed the survey. The survey was conducted nationwide, covering a wide range of metropolitan and provincial areas.

### 2.4. Study Design

A cross‐sectional study was conducted using a structured online questionnaire.

### 2.5. Sample Size Determination and Sampling Technique

As of March 2023, the Korean Veterinary Medical Association reported 6,938 licensed veterinarians engaged in companion animal care [7]. This study obtained responses from a final sample of 114 companion animal veterinarians, representing approximately 1.64% of this population. While achieving a 95% confidence level with a narrow +‐5% margin of error would typically require a sample size of around 370; this study was designed as an exploratory assessment rather than a confirmatory epidemiological survey. Given practical constraints such as time, cost, and limited accessibility to veterinarians, the final sample of 114 was considered sufficient to capture broad patterns and generate meaningful insights into ophthalmic examination practices, referral patterns, and challenges in companion animal clinical settings in South Korea. This approach is statistically justified as it maintains a 90% confidence level with an estimated margin of error of +‐7.7%, which is well within the acceptable range for preliminary social and behavioral science research [8]. Consequently, the findings provide a foundational understanding to support future research and policy development, consistent with the study′s goal of identifying trends and generating hypotheses for future in‐depth studies.

### 2.6. Sampling Procedure

The survey was conducted nationwide, encompassing most regions, but the majority of responses came from metropolitan areas such as Seoul and Gyeonggi, where veterinary clinics are generally more active on social media. As the survey was administered in Korean and online, no language adaptation or translation was required. A pretest was conducted with three Korean veterinarians and six health tech industry professionals to ensure clarity and relevance.

### 2.7. Method of Data Collection

Data were collected via Google Forms between April 23 and May 6, 2024. The survey originally included 16 questions; however, five items related to telemedicine and equipment purchasing intentions were excluded from the analysis. As a result, 11 questions were used in this study. The final version of the questionnaire is presented in Appendix A, which is included at the end of this document.

### 2.8. Data Management and Analysis

Responses were stored and coded using Microsoft Excel and analyzed with STATA, version 17. Descriptive statistics, including frequencies and percentages, were used to summarize the data. Logistic regression was initially performed to identify factors influencing the provision of ophthalmic and fundus examinations; however, no statistically significant variables were identified, and thus regression results were omitted from the findings.

## 3. Results and Discussion

### 3.1. Basic Characteristics

A total of 114 veterinarians participated in the survey. The majority were male (71.9%), with female respondents accounting for 28.1%. Most participants were in their 30s (56.1%) or 40s (32.5%), with smaller proportions in their 20s (4.4%) and 50s (7.0%). In terms of education, 74.6% held a bachelor’s degree, while 25.4% had obtained a master’s degree or higher. Most respondents had 1–10 years of clinical experience (73.7%) and worked in primary clinics (81.6%). Most participants were private practice veterinarians (64.9%). Geographically, 65.8% were based in metropolitan areas such as Seoul, Incheon, or Gyeonggi Province.

Referral patterns to specialized eye care facilities varied depending on respondents’ affiliations with university hospitals or specialized clinics. These characteristics contextualize the subsequent findings, as presented in Table 1.

**Table 1:** Demographic and professional characteristics of survey respondents (*N* = 114).

Variables	Category	No. of respondents	Frequency (%)	95% CI
Sex	Male	82	71.9	63.1–79.4
	Female	32	28.1	20.6–36.9

Age (yr)	20’s	5	4.4	1.9–9.9
	30’s	64	56.1	47.0–64.9
	40’s	37	32.5	24.6–41.5
	50’s	8	7.0	3.6–13.2

Educational status	Bachelor’s degree	85	74.6	65.9–81.7
	Master’s degree or higher	29	25.4	18.3–34.1

Clinical experience	1–5 yrs	37	32.5	24.6–41.5
	6–10 yrs	47	41.2	32.6–50.4
	11–15 yrs	16	14.0	8.8–21.6
	16–20 yrs	6	5.3	2.4–11.0
	21–25 yrs	7	6.1	3.0–12.1
	26–30 yrs	0	0	0.0–3.3
	Over 30 yrs	1	0.9	0.2–4.8

Type of animal clinics	Primary care clinic	93	81.6	73.5–87.6
	Referral or specialty hospital	21	18.4	12.4–26.5

Employment status	Private practitioner	74	64.9	55.8–73.1
	Salaried veterinarian	40	35.1	26.9–44.2

Location of animal clinics	Metropolitan area (Seoul, Incheon, and Gyeonggi)	75	65.8	56.7–73.9
	Nonmetropolitan area (other than metropolitan area)	39	34.2	26.1–43.3

Referral for more specialized eye care^∗^	Often	19	16.7	9.9–23.5
	Sometimes	75	65.8	57.1–74.5
	Rarely	10	8.8	3.6–14.0
	Never	3	2.6	0.0–5.5
	Do not know	5	4.4	0.6–8.2
	Not applicable (respondent works in an ophthalmic specialized hospital and a university‐affiliated hospital)	2	1.7	0.0–4.1

^∗^To specialized hospitals and university‐affiliated hospitals.

### 3.2. Provision of Ophthalmic Examination in Veterinary Clinics

#### 3.2.1. General Ophthalmic Services

More than 80% of respondents (*n* = 101) reported providing ophthalmic examinations, which included vision testing, anterior segment evaluation, and/or fundus examination (Table 2).

**Table 2:** Availability of general ophthalmic examination services among respondents (*N* = 114).

Variables	Category	No. of respondents	Frequency (%)	95% CI
Ophthalmological examination^∗∗^	Yes	101	88.6	82.4–94.8
	No	13	11.4	1.9–20.9

^∗∗^Includes cases even if only partial provision is provided, such as only vision examination and only anterior segment (cataract, intraocular pressure, etc.) examination.

#### 3.2.2. Fundus Examinations

Approximately one‐third of respondents (*n* = 35) reported offering fundus (posterior segment) examinations (Table 3), a rate that was substantially lower than the proportion of veterinarians offering anterior segment evaluations (Table 2). Among those not offering fundus examinations (*n* = 64), the most cited reason was the high cost of necessary equipment (70.3%), followed by limited diagnostic confidence (48.3%) and perceived low clinical need (28.1%) (Table 3).

**Table 3:** Availability of fundus examination services and reasons for nonprovision (*N* = 101; *n* = 64).

Variables	Category	No. of respondents	Frequency (%)	95% CI
Fundus examination^∗∗∗^ (among 101 respondents)	Yes	35	34.7	20.0–49.4
	No	64	63.4	50.0–76.8
	Don’t know	2	1.9	0.0–4.4

Reasons for not providing fundus examinations (among 64 respondents/including duplicates)	High cost of fundus examination equipment	45	70.3	56.9–83.7
	Difficulty of diagnosing fundus diseases	28	48.3	29.8–66.8
	Insufficient need for fundus examinations	18	28.1	7.3–48.9
	Other	3	4.7	0.0–28.7

^∗∗∗^Observe the condition of the fundus (posterior segment of eye) through the pupil.

A logistic regression analysis was conducted to explore factors associated with the provision of eye and fundus examinations, but no statistically significant associated facors were identified. This suggests that demographic or educational variables may not be strongly associated with the likelihood of offering these services.

## 4. Conclusions

This study investigated the current status of ophthalmic examination services in South Korea, focusing on their availability, referral practices, and barriers to provision. Given the limited research on veterinary clinical service delivery [9], these findings contribute valuable insights into the structure and challenges of animal healthcare.

Compared to anterior segment evaluations, fundus examinations were performed by far fewer respondents (*n* = 35)—by only about one‐third of the total. This rate is significantly lower than that reported in similar studies conducted in the United States [10], indicating a potential gap in diagnostic capabilities for posterior segment eye diseases in South Korean veterinary practice.

As the pet population ages, there is a growing need to monitor and manage chronic diseases that may affect the fundus. The main barriers identified were high equipment costs, lack of diagnostic confidence, and low perceived need. To address these, it is important to raise awareness of affordable diagnostic tools such as direct ophthalmoscopes priced under $40 and smartphone‐compatible indirect lenses available for under $100. For example, while an original manufacturer‐produced Volk 20D lens costs approximately $432, comparable alternatives are available online for as little as $23.

Beyond addressing the economic barriers associated with equipment costs, improving veterinarians’ diagnostic confidence will require structured education and training. Fundus examination skills should be integrated into the core curriculum of veterinary schools and reinforced through continuing education. Various methods for fundus examination are available, each with distinct advantages and limitations. A comparative overview of fundus examination methods is provided in Appendix A1 to support a more comprehensive understanding of practical options. Despite the fact that teleophthalmology may be an effective and convenient support tool in some scenarios, it must be recognized that there are some limitations, including a steep learning curve, difficulties getting diagnostic‐quality images, and a lack of 3D visualization.

Finally, some respondents noted a low perceived clinical need for fundus examinations. This may reflect pet owners’ limited awareness rather than an actual lack of clinical necessity. As societal expectations around animal welfare and quality of life continue to evolve, clinical need for comprehensive diagnostic services, including ophthalmology, is expected to rise. The findings of this study may serve as a foundation for improving veterinary education, informing service delivery strategies, and guiding the development of more accessible and effective diagnostic tools for companion animal care.

## Disclosure

This research was carried out as part of their employment at LabSD, Inc., which provided general support and resources but did not influence the study’s design, data collection, analysis, interpretation, or publication. Following the completion of the study, LabSD, Inc. expanded its services into the veterinary field to address unmet healthcare needs identified through this research. While this subsequent involvement may present a potential conflicts of interest, it is driven by the company’s broader mission to improve veterinary healthcare through technology.

## Conflicts of Interest

The authors declare no conflicts of interest.

## Author Contributions

Conceptualization: Jae‐Won Kim. Methodology: Jae‐Won Kim. Investigation: Jae‐Won Kim and Kyeongmin Kim. Data Curation: Jae‐Won Kim. Formal analysis: Jae‐Won Kim. Writing–original draft: Jae‐Won Kim. Writing–review and editing: Jae‐Won Kim, Holden Yoon Seung Kim, and Kyeongmin Kim. Visualization: Jae‐Won Kim. Supervision: Jae‐Won Kim and Holden Yoon Seung Kim. Project administration: Kyeongmin Kim.

## Funding

The authors received no specific funding for this work.

## Data Availability

The data that support the findings of this study were provided by LabSD, Inc. Restrictions apply to the availability of these data, which were used under license for this study. Data are available from LabSD, Inc. or its designated authority with the permission of LabSD, Inc.

## Appendix A Survey Questionnaire for Veterinary Clinics in Korea

1. What is your sex?
a. Male
b. Female
2. Which of the following age groups do you belong to?
a. 20 s
b. 30 s
c. 40 s
d. 50 s
e. 60 years or older
3. What is your highest level of education?
a. Bachelor’s degree (DVM or equivalent)
b. Master’s degree
c. Doctorate (PhD, specialist degree, etc.)
4. How many years have you been practicing veterinary medicine?
(Open‐ended: Enter number of years. If less than 1 year, enter “1”)
5. What is your current professional status?
a. Private practitioner (including co‐owner)
b. Salaried veterinarian
6. Where is your hospital located?
(Open‐ended: Provide up to city/province and district/county)
7. Does your hospital provide ophthalmologic examinations?
(Ophthalmologic examinations include vision testing, anterior segment evaluation, and fundus examination.)
a. Yes
b. No
8. If yes, how many ophthalmologic examinations are performed per week at your hospital?
(Open‐ended: If unknown, respond “I do not know”)
9. Does your hospital perform fundus examinations?
(Fundus examination is defined as the evaluation of the retina through the pupil.)
a. Yes
b. No
10. If your hospital does not provide fundus examinations, what are the reasons?
(Select all that apply.)
a. Equipment is expensive
b. Fundus diseases are difficult to interpret
c. There is no clinical need
d. Other (please specify)
11. How often do you refer patients to ophthalmology clinics or higher‐level hospitals for specialized eye care?
a. Often
b. Sometimes
c. Rarely
d. Never
e. Do not know
f. Prefer not to answer
12. What type(s) of fundus examination equipment does your hospital currently use?
(Multiple selections allowed)
a. Direct ophthalmoscope (observation only; no image capture)
b. Table‐top fundus camera
c. Handheld fundus camera
d. Do not know
13. What aspects of your current fundus examination equipment would you like to see improved?
(Multiple selections allowed)
a. Very satisfied with current equipment
b. No specific complaints
c. Image quality is insufficient
d. Purchase cost is high
e. Maintenance cost is high (e.g., consumables)
f. Operation is inconvenient or difficult
g. Limited image storage capacity
h. No photo‐capturing function
i. Do not know
14. If a fundus camera with the following specifications were available, would your hospital consider purchasing it?
(Specifications: handheld, ≥ 55° field of view, high‐resolution imaging, mid‐to‐low market price)
a. Yes
b. No
15. What would be your top priority when purchasing a fundus camera?
a. Price
b. Ease of use
c. Recommendation from a trusted peer or colleague
16. If a specialist‐led remote fundus image interpretation service were offered as a paid option with the purchase of a fundus camera, would you be interested in using it?
a. Yes
b. No

**Table Appendix A1:** Comparison of fundus examination methods.

Method	Description	Advantages	Disadvantages	Cost range	Example devices
Direct ophthalmoscope (DO)	Direct visualization of the retina by shining light directly onto it. Produces an upright image.	Relatively inexpensive; allows close observation with high magnification.	High difficulty; requires training and experience. Extremely narrow field of view, mainly limited to optic disc examination.	$20–$600	Heine Mini 3000, Welch Allyn PocketScope
Monocular indirect ophthalmoscope (MIO)	Indirect visualization of the retina through a lens. Viewed with one eye (monocular), producing a flat, 2D inverted image.	Fast screening; lightweight and simple equipment; relatively inexpensive; easier for beginners to learn.	Best suited for examining the central fundus; moderate learning curve; somewhat challenging to use (less than BIO).	$100–$600+	PanOptic by Welch Allyn
Binocular indirect ophthalmoscope (BIO)	Indirect visualization through a lens using both eyes, allowing for 3D depth perception. Produces an inverted image.	Enables detailed fundus examination, including peripheral retina; good depth perception.	Requires head‐mounted equipment; heavier and needs setup; high learning curve; expensive.	$200–$1000+	Keeler vantage plus
Conventional (table‐top) fundus camera	Stationary camera designed for retinal imaging. Provides a wide field of view depending on lens configuration. Produces upright images.	High‐resolution digital images; allows digital magnification and objective documentation; records full retina including periphery; some models support OCT and 3D imaging.	High cost; nonportable; requires dedicated space; requires user skill in adjusting focus and lighting; time‐consuming; patient cooperation needed.	$1000–$30,000+	Canon CR‐2, topcon TRC‐NW8
Portable fundus camera	Handheld device for capturing retinal images.	Enables quick imaging; requires minimal patient positioning; relatively inexpensive; highly portable.	May offer lower image quality and resolution compared to table‐top models.	$500–$5000+	Welch Allyn RetinaVue 700 imager retinal camera
Smartphone with lens attachment (e.g., 20D, 72D, 90D, and more)	Uses a smartphone with a lens adapter to capture fundus and retinal images.	Highly portable and low cost (only lens purchase required); convenient for field use.	Requires high operator skill; image quality and stability depend on smartphone hardware; difficult to obtain consistent results.	$20–$500+	Volk lenses and D‐eye
